# Metabolomics approach reveals urine biomarkers and pathways associated with the pathogenesis of lupus nephritis

**DOI:** 10.22038/ijbms.2019.38713.9178

**Published:** 2019-11

**Authors:** Shiva Kalantari, Saeed Chashmniam, Mohsen Nafar, Zahra Zakeri, Mahmoud Parvin

**Affiliations:** 1 Chronic Kidney Disease Research Center, Shahid Beheshti University of Medical Sciences, Tehran-Iran; 2 Department of Chemistry, Sharif University of Technology, Tehran, Iran; 3 Urology-Nephrology Research Center, Shahid Beheshti University of Medical Sciences, Tehran, Iran; 4 Department of Rheumatology, Shahid Labbafinejad Medical Center, Shahid Beheshti University of Medical Sciences Tehran, Iran; 5 Department of Pathology, Shahid Labbafinejad Medical Center, Shahid Beheshti University of Medical Sciences Tehran, Iran

**Keywords:** Biomarker, Lupus nephritis, Metabolomics, Non-invasive diagnosis, Nuclear Magnetic – Resonance, Urinary metabolites

## Abstract

**Objective(s):**

lupus nephritis (LN) is a severe form of systemic lupus erythematosus (SLE) with renal complications. Current diagnosis is based on invasive renal biopsy and serum antibodies and complement levels that are not specific enough. The current study aims to identify new biomarker candidates for non-invasive diagnosis of LN and explore the pathogenic mechanisms that contribute to renal injury.

**Materials and Methods:**

A metabolomics approach using 1H-nuclear magnetic resonance (1H-NMR), was developed for comparison of urine metabolic profile of 14 LN patients, 10 SLE patients, and 11 healthy controls (HCs). Differential biomarker candidates were identified by using multivariate modeling, and their diagnostic accuracy was evaluated by receiver operating characteristic analysis (ROC).

**Results:**

Three metabolites were common in differentiating all three groups including beta-alanine, 2,2-dimethylsucssinic acid, and 3,4-Dihydroxyphenylacetaldehyde and suggested as a diagnostic panel for LN with AUC of 0.89, sensitivity of 81 %, and specificity of 100 %. Complementary analyses on pathways indicated that nicotinate and nicotinamide metabolism is the most important perturbed pathway in LN.

**Conclusion:**

Metabolomics is a useful tool for identification of biomarkers with the ability to diagnose LN patients and predict perturbed pathways responsible for renal injury.

## Introduction

Lupus nephritis (LN) is an organ-specific complication of systemic lupus erythematosus (SLE), with severe mortality and morbidity (1, 2). About 50–60% of patients with SLE end up with renal abnormalities with combinations of symptoms such as edema, proteinuria, hypertension, urinary sediment abnormalities, and reduced renal function (3, 4). Pathogenesis of LN seems to depend on antibody binding to multiple intrarenal autoantigens or formation of circulating immune complexes containing autoantibodies and their deposition on different parts of the glomeruli (e.g., mesangium, subendothelial, and/or subepithelial space near the glomerular basement membrane) (5, 6), nevertheless, there is still a lack of sufficient knowledge of what immune-pathological pathways are involved (7, 8). 

Kidney biopsy is considered the gold standard for diagnosis, staging (using the International Society of Nephrology/Renal Pathology Society (ISN/RPS) Classification)(9), prediction of renal outcomes, and decision-making for treatment (10). However, the limitations, such as invasiveness, performing a serial biopsy for monitoring patients, and potential complications of biopsy, affect its application (11, 12). Therefore, searching for new specific noninvasive biomarkers of LN is of great importance. 

Metabolic profiling (metabolomics) that could detect and quantify low molecular weight molecules (metabolites) in biological fluids has great value in identifying diagnostic biomarkers in different diseases, including renal diseases (13-17). Nuclear magnetic resonance (NMR) spectroscopy, liquid chromatography or gas chromatography coupled with tandem mass spectrometry (LC-MS/MS and GC-MS/MS, respectively) are the most widely used analytical techniques for metabolomics studies; the former is privileged as it is less expensive, requires minimal sample preparation, and is rapid (12). This study aimed to explore whether NMR-derived urine metabolomics would reveal a specific signature of LN and thereby, suggest novel biomarker candidates for predicting the renal complications of SLE, as well as understanding metabolic pathways involved in the pathogenesis of LN.

## Materials and Methods

***Subjects and participants***

The urine samples used in this study were collected from SLE and LN patients attending Labbafinejad Medical Center, Tehran, Iran. Urine samples were collected from fourteen patients with LN (11 females, 3 males, mean age 37.7±9.26 years), ten patients with SLE (8 females, 2 males, mean age 40.6±11.5 years), and eleven healthy control subjects (7 females, 4 males, mean age 39.43±14.34 years). Inclusion criteria for LN patients were confirmation of kidney involvement in SLE by a pathologist based on kidney biopsy and proteinuria in 24 hr urine. SLE patients were included in the study if their rheumatological tests were positive for SLE (e.g., decreased serum complement factors, positive antinuclear antibodies (ANA), and positive anti-double-stranded DNA antibody (anti-dsDNA)) without renal injury symptoms. Healthy subjects had normal clinical tests and had no history of diseases. The demographics and clinical characteristics of SLE and LN patients are tabulated in Table 1. All experimental protocols of this study were approved by the Chronic Kidney Disease Research Center Ethics Committee, Shahid Beheshti University of Medical Sciences, Tehran, Iran, and informed consent was obtained from all participants. 

***Sample collection and preparation for NMR spectroscopy***

Urine samples were collected from all subjects (morning, second pass) in sterile urine cups and centrifuged at 3000 rcf for 20 min at 4 ^°^C. The casts were removed, and the supernatant was subjected to ultrafiltration by Amicon Ultra-15 centrifugal filter units with a 3 kDa cutoff (Millipore, Billerica, MA, USA) to filter the proteins. The flow-through fractions were stored at −hr °C and applied for metabolomics analyses. At the time of NMR measurement, urine samples were thawed at room temperature, vortexed and the aliquots of 450 μl of urine samples were added to 60 μl of a buffer containing 300 mM potassium phosphate buffer (KH2PO4), 0.2 % 3-(trimethylsilyl)propionic acid sodium salt (TSP), and 20 % D2O. TSP was used for locking and chemical shift referencing. The total mixture of samples and buffer was then moved into a 5 mm NMR tube (Sigma Aldrich, RSA), and subjected to NMR spectroscopy.

***NMR Measurements and data processing***

The NMR experiments were performed at 300 K on a Bruker DRX 500 MHz NMR spectrometer.

One-dimensional CPMG and diffusion edited ^1^H-NMR spectra were recorded on all urine samples using the Carr–Purcell–Meiboom–Gill pulse sequence. The parameters used for 1D CPMG pulse sequence were as follows: data points: 32 K, flip angle of radiofrequency pulse: 90 °, relaxation delay : 2.5 sec, the total T2 filtering time was set at 43 msec, number of scans: 154, recycle delay was 1.5 sec, window function: exponential and line broadening: 0.3 Hz. The processing and reduction of NMR data were done using PROMETAB software (version prometab_v3_3) (18) in MATLAB (version 6.5.1, The MathWorks, Cambridge, UK) in the chemical shift region δ 0.2–10.0 ppm. 

The spectra were then binned into 0.02 ppm integrated spectral buckets. The chemical shift region of water, including δ 4.2–5.2 ppm, was excluded from the analysis. The binned spectral data were obtained from PROMETAB after normalization, which was performed by the quantile normalization method. The resulting data matrices were then exported into SIMCA (Version 14.0, Umetrics, Umea, Sweden) for further statistical analyses.

***Pattern recognition analysis and multivariate modeling***

Before pattern recognition and modeling, the data were mean-centered and scaled using unit variance in which identical weight was given to all variables. For determining the differences among LN and control groups (i.e., HC and SLE), pair-wise multivariate analysis was performed for LN vs HCs and LN vs SLEs. Unsupervised principal component analysis (PCA) was first carried out on the dataset for pattern recognition, viewing the data structure, and outlier detection. The residual and absolute outliers were recognized by this method and excluded from the analyses. To further demonstrate the differences between the different groups, supervised orthogonal projection latent to structure discriminant analysis (OPLS-DA) was employed to help to identify potential discriminatory metabolites. Model validation was done using repeated 7-fold internal cross-validation. The reliability of the models was further validated by the permutation tests (n=950). The candidate bines (i.e., chemical shifts) were identified from the scores of variable importance on projection (VIPs>1) in OPLS-DA models. The candidate chemical shifts were corresponded to related metabolites by using databases (e.g., human metabolome database (HMDB), biological magnetic resonance bank (BMRB)) and literature reports. The receiver operating characteristic (ROC) analysis was carried out to evaluate each candidate metabolite biomarker. The area under the curve (AUC) from ROC analyses was computed using the SPSS software package (version 24, IBM).

***Pathway analysis***

Using the IMPaLA web tool (http://impala.molgen.mpg.de), we identified the pathways associated with renal injury in LN patients. The corrected *P*-value < 0.05 was considered significant in this analysis. Then, the most important pathway that was identified by IMPaLa, was visualized using the MetScape plugin  (http://metscape.ncibi.org/) in Cytoscape (http://www.cytoscape.org/) (18). The genes and other compounds that were related to the target metabolites in this pathway were visualized as a network. The MetScape tool provides a platform for the visualization and interpretation of experimental data from genes and metabolites in the context of human metabolism. This plugin uses KEGG and EHMN databases. 

## Results

***Multivariate analysis and systematic differences between groups***

**Figure 1:**
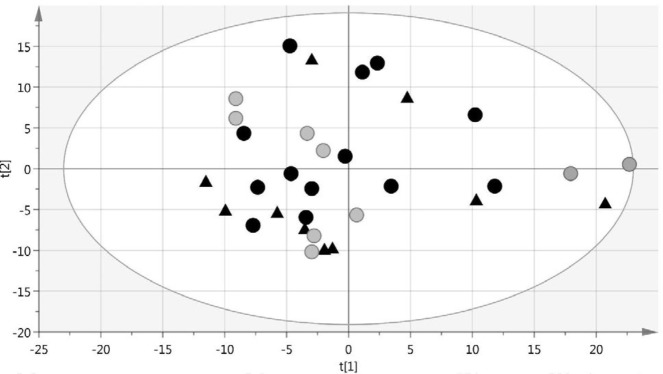
PCA score plot of observations after outlier exclusion. Black circles represent LN patients, open circles represent SLE patients, and black triangles represent healthy controls. LN: lupus nephritis; SLE: systemic lupus erythematosus; PCA: principal component analysis

**Figure 2:**
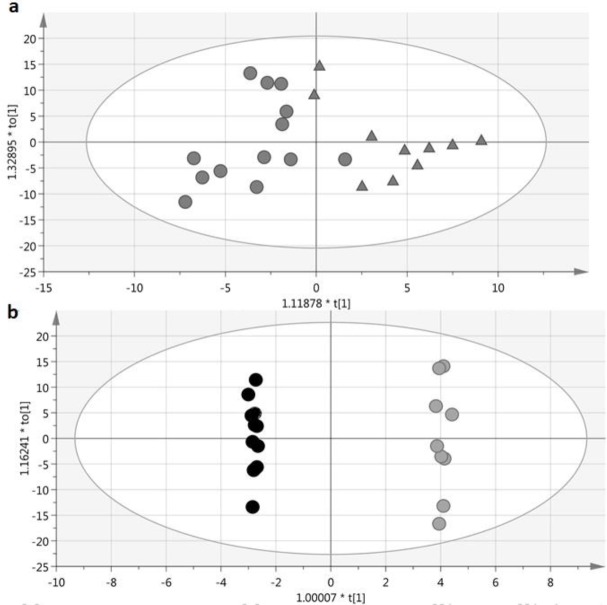
OPLS-DA score plots derived from 1H-NMR of urine. (a) LN (circles) as compared with the healthy control (triangles); (b) LN (black circles) as compared with the SLE patients (open circles)

LN: lupus nephritis; SLE: systemic lupus erythematosus; OPLS-DA: orthogonal projection latent to structure discriminant analysis

Principal Component Analysis (PCA) was performed on data matrices, which were obtained from NMR spectra, and the corresponding score plot showed an absolute outlier from the LN group (Figure S1). To identify residual outliers, DModX line plot was drawn, and two other outliers from HC and SLE groups were identified (Figure S2). The three identified outliers were excluded from the analysis. Figure 1 shows the score plot of PCA after exclusion of outliers. There was no clear clustering in this plot, and all the remaining subjects were in the defined confidence interval (Hotelling’s T2 = 0.95%). Pairwise supervised modeling of OPLS-DA was performed to discriminate LN patients from SLE patients and HCs. The OPLS-DA models demonstrated clear separation between LN and HCs (Figure 2-a), LN, and SLE (Figure 2-b). Parameters of the models are summarized in Table 2. Model validation was done using repeated 7-fold cross-validation and 950 times permutation tests. These model evaluations confirmed that the models are valid since the AUCs of the models were high, and Q2 values of permutations were lower than the original models (Table 2). 

***Differential candidate biomarkers***

**Figure 3:**
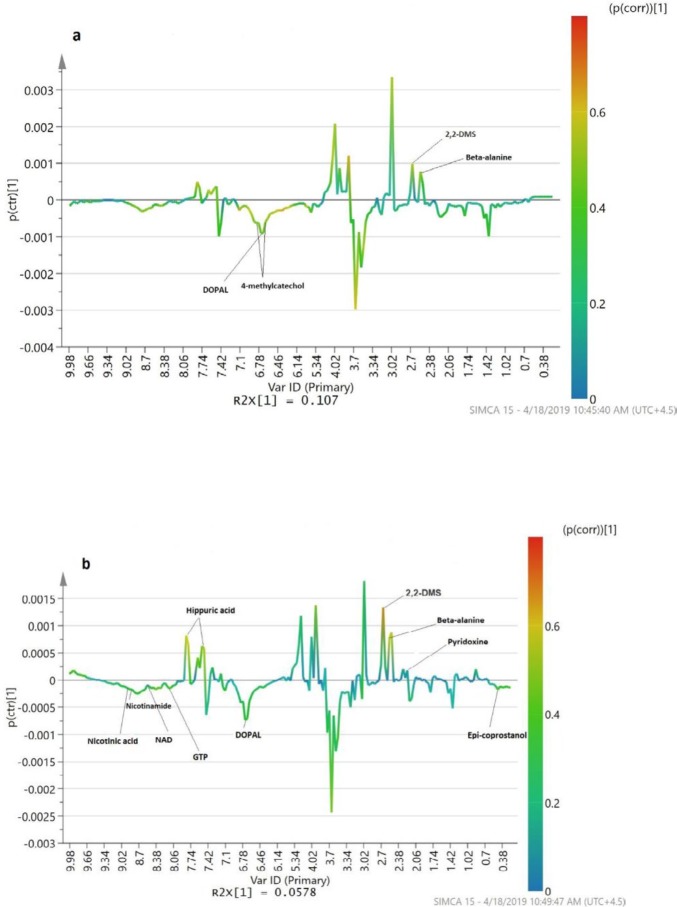
S-line plot of diagnostic models and candidate biomarkers. (a) LN vs HC; (b) LN vs SLE

LN: lupus nephritis; HC: healthy control; SLE: systemic lupus erythematosus

Metabolic features (chemical shifts) that were significant in each multivariate model and had fold changes > 1.5 were considered as diagnostic candidates and were identified. Candidate metabolic biomarkers that were underrepresented in the urine of LN patients in comparison with HCs were 4-methylcatechol and 3,4-Dihydroxyphenylacetaldehyde (DOPAL), while 2,2-dimethylsucssinic acid and beta-alanine were overrepresented (Table 3). Underrepresented candidate metabolites in the urine of LN patients in comparison with SLE patients were nicotinamide ribotide (NMN), nicotinamide, guanosine triphosphate (GTP), nicotinamide adenine dinucleotide (NAD), nicotinic acid, and DOPAL, versus six other candidate metabolites that were overrepresented in LN patients including epi-coprostanol, 2,2-dimethylsucssinic acid, beta-alanine, pyridoxine, hippuric acid, and anthranilic acid (Table 3). The candidate biomarkers on the S-line plot of diagnostic models are shown in Figures 3-a and 3-b.

***Diagnostic evaluation of the biomarkers***

To evaluate the diagnostic accuracy of identified candidate biomarkers, ROC analysis was performed, and AUC of each candidate metabolite was calculated (Table 4). Beta-alanine was the best discriminator between LN and HCs with a sensitivity of 90%, specificity of 100%, and AUC of 0.9. 2,2-dimethylsucssinic acid was the best discriminator between LN and SLE patients with a sensitivity of 80%, specificity of 100%, and AUC of 0.88. Three metabolites that discriminate LN patients from both SLE group and HCs were beta-alanine, 2,2-dimethylsucssinic acid, and DOPAL. A panel composed of these three biomarker candidates or composed of 2,2-dimethylsucssinic acid and DOPAL had the same AUC, sensitivity, and specificity, which improve the total diagnostic accuracy of renal injury in LN patients compared with single biomarkers.

***Pathways associated with renal injury in LN patients***

**Figure 4.**
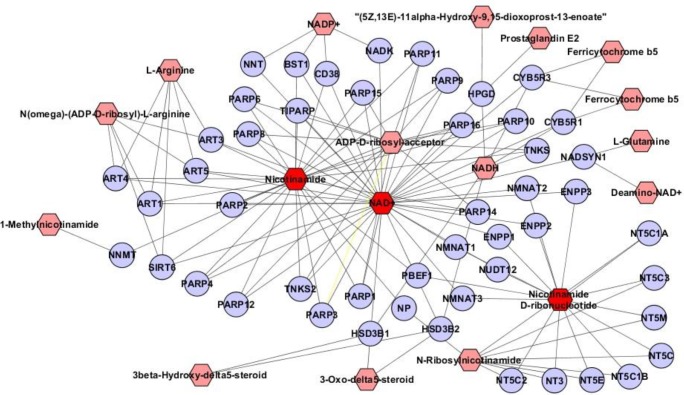
The subnetwork of target metabolite in the "nicotinamide metabolism" pathway, their related genes, and other compounds that are involved in this pathway. Red octagons represent target metabolites in our dataset, pink octagons represent other related compounds in this pathway, and circles represent related genes that contribute to this pathway

To identify the impaired pathways in the pathogenesis of LN, the differential metabolites among the three groups were subjected to pathway analysis by using the IMPaLA web tool. Twenty four pathways were significant, with *P*-value<0.05 (Table 5). Nicotinate and nicotinamide metabolism pathway (*P*-value = 0.000225), DNA damage recognition in global genome nucleotide excision repair (GG-NER) (*P*-value= 0.00156), and degradation of AXIN (*P*-value= 0.00156) were the top three impaired pathways in LN patients. To visualize the relationship of target metabolites, their related genes, and other compounds in nicotinate and nicotinamide metabolism as the most important pathway in LN pathogenesis, the MetScape plugin in Cytoscape was used (Figure 4). The names of related genes with the target metabolites (i.e., nicotinamide, NMN, and NAD) are summarized in Table S1. These genes could be the potential targets for future analysis for the regulation of this pathway. 

## Discussion

In the present study, we have demonstrated the potential of the^ 1^H-NMR-based metabolomic technique for non-invasive diagnosis of LN patients from SLE patients and HCs based on their characteristic urine metabolite profiles. To the best of our knowledge, this is the first pilot study on urine metabolomics for LN and its comparison with two HC and disease control (i.e., SLE) groups. The other metabolomics studies on LN have focused on discrimination between membranous LN, proliferative LN, and focal segmental glomerulosclerosis (10), or studied the serum metabolic profile of LN, SLE, and HC subjects (12, 15). The advantage of the present study is the use of urine metabolic profile as the microenvironment of injury and non-invasive approach for diagnosing the renal damage in LN patients. 

The study revealed a wide range of differential metabolites in LN patients of which combination of 2,2-dimethylsucssinic acid and DOPAL with or without beta-alanine, provides the best diagnostic accuracy for development of renal injury in these patients. The metabolic signature of LN indicated perturbation of several pathways, including nicotinate and nicotinamide metabolism and pathways related to DNA repair.

Beta-alanine is one of the two amino acids (besides histidine) that produce carnosine (19). Scavenging reactive oxygen species is the possible role of carnosine in the kidney, which has reno-protective effects in diabetic nephropathy (20). There is evidence that carnosine is synthesized and metabolized in the kidney, and its metabolism plays an essential role in maintaining normal kidney function (21). Significant decreased beta-alanine urinary excretion in LN patients in comparison with SLE and HC subjects (with a fold change of 2.4 and 3.5, respectively) might be due to decreased carnosine content of kidneys in LN patients, which may make their kidneys susceptible to insults of oxidative stress products. 

2,2-dimethylsuccinate (2,2-DMS) is one of the plasma and urine metabolites that can specifically inhibit the Na +-dependent dicarboxylate transporters such as NaDC-3 and NaDC-1, which express on basolateral and luminal plasma membranes of renal proximal tubule cells, respectively (22, 23). Krebs cycle intermediates such as succinate are imported from urine by theses transporters to be used as energy substrates by proximal tubular cells (24, 25). Since the affinity of these dicarboxylate transporters to 2,2-DMS is higher than succinate (23), and hence, importing 2,2-DMS could be faster than succinate, one can postulate that, decreased level of 2,2-DMS in the urine of LN patients might be due to the higher activity of these transporters in the tubular cells of nephritis patients. The activity of dicarboxylic transporters imports the intermediates of the Krebs cycle to produce more energy. The proliferation of endocapillary and extra capillary cells, known histopathologic characteristics of the proliferative form of LN (class IV), needs energy, which can support the reason for this energy demand. Increased urinary level of nicotinamide, NAD, and nicotinamide ribonucleotide (NMN) in LN patients that are involved in “vitamin B3 (nicotinate and nicotinamide) metabolism” and linked to fuel utilization and energy metabolism, also support the hypothesis of increased activity of dicarboxylate transporters and decrease the level of 2,2-DMS. 

DOPAL, 3,4-dihydroxyphenylacetaldehyde, is a very reactive aldehyde and toxic metabolite of dopamine, which results from the activity of monoamine oxidase (26, 27). Protein modifications via formation of Schiff base is an essential mechanism of toxicity for DOPAL, which can result in inhibition of enzyme activity and loss of function for cellular proteins (28, 29). The target proteins for modification by DOPAL are chaperones, detoxification enzymes, metabolic proteins, ER proteins, and inflammatory proteins, the modifications of which result in oxidative stress, increased presence of toxic metabolites, and protein aggregation (30). Since oxidative stress conditions and inflammation have major roles in the pathogenesis of LN (31), the relationship of DOPAL with these conditions may explain the higher level of this toxic metabolite in the urine of LN patients.

DNA damage recognition in GG-NER was the important pathway after nicotinamide metabolism that is impaired in LN patients. A recent study by Souliotis *et al*. (32) confirms severe DNA damage in patients with active LN, which results in higher DNA damage levels than those with quiescent disease (32).

Degradation of AXIN, a component of the beta-catenin destruction complex and modulator of Wnt signaling (33), is also another impaired pathway in LN. Activation of Wnt signaling in LN patients resulting from increased mRNA expression of axin-2 in renal tissues of these patients was reported by Wang *et al*. (33). Overrepresentation of degradation of axin in our dataset, which results in activation of Wnt signaling confirms the role of this pathway in pathogenesis of LN. 

On testing this result, the multivariate discriminant models obtained considerable predictive values (Q2) and great sensitivity and specificity to diagnose LN (Table 2).

Furthermore, when the three biomarkers that were significant in both OPLS-DA models (LN vs SLE and LN vs HC) and with highest AUC were combined as a panel, the ability to diagnose LN was enhanced compared with individual biomarkers. In the present study, we found that the highest AUC value was obtained when beta-alanine, 2,2-dimethylsucssinic acid, and DOPAL were combined. The AUC value of this combined biomarker pattern was 0.89, which beside sensitivity of 81% and specificity of 100% indicates excellent diagnostic accuracy. 

**Table 1:** Demographics and clinical characteristics of systemic lupus erythematosus (SLE), lupus nephritis (LN) patients, and healthy controls

Characteristics	LN patients	SLE patients	Healthy controls
Number of samples	14	10	11
Age (years), Mean ± SD	37.7 ± 9.26	40.6 ± 11.5	39.43 ± 14.34
Gender (F/M)	11/3	8/2	7/4
Albumin (g/l)	2.37 ± 0.64	-	-
Creatinine (mg/dl)	1.4 ± 0.69	0.99 ± 0.1	Normal
eGFR (m/min/1.73 m^2^)	63.7 ± 30.8	70 ± 8.5	Normal
FBS (mg/dl)	111 ± 36.5	< 90	< 90
BUN (mg/dl)	32.75 ± 25	12.27 ± 13	-
Uric acid (mg/dl)	6.4 ± 1.5	-	-
Triglyceride (mg/dl)	236.7 ± 166.6	-	104 ± 27.3
Cholesterol (mg/dl)	204.6 ± 38.7	-	197.5 ± 3.8
HDL (mg/dl)	41.33 ± 19.6	-	49.3 ± 7.5
LDL (mg/dl)	140 ± 17.2	-	127.3 ± 33.3
ESR (mm/h)	41.5 ± 11.2	25.5 ± 8.5	-
CRP (mg/l)	3.7 ± 2.6	1.8 ± 2	-
Proteinuria (mg/24h)	3088.75 ± 908	< 150	< 150
RF (lu/ml)	21.5 ± 15.8	3 ± 10	-
C3 (mg/dl)	93 ± 59	90.5 ± 15	-
C4 (mg/dl)	20.2 ± 15	13.5 ± 15	-

LN: lupus nephritis; SLE: systemic lupus erythematosus; F: female; M: male; LDL: low-density lipoprotein; HDL: high-density lipoprotein; ESR: erythrocyte sedimentation rate; CRP: C-reactive protein; BUN: blood urea nitrogen; FBS: fasting blood sugar; RF: rheumatoid factor; eGFR: estimated glomerular filtration rate

Quantitative data are presented as mean±SD

**Table 2:** Parameters of cross-validated OPLS-DA applied to spectra of urine samples between groups (LN vs HCs and LN vs SLE)

Parameters	LN vs HC	LN vs SLE
**R2X (cum)**	**0.388**	**0.932**
**R2Y (cum)**	**0.681**	**0.999**
**Q2 (cum)**	**0.101**	**0.805**
**AUC**	**0.67**	**1**
**Sensitivity (%)**	**70%**	**100%**
**Specificity (%)**	**70%**	**100%**
**R2 of permutated model**	**0.45**	**0.988**
**Q2 of permutated model**	**-0.4**	**-0.66**

**Table 3:** Key observed metabolic differences between lupus nephritis (LN) and healthy control (HC) and between LN and systemic lupus erythematosus (SLE)

**Metabolite**	**Chemical shift**	**LN vs SLE**			**LN vs HC**		
		**Variation**	**VIP**	**Fold change**	**Variation**	**VIP**	**Fold change**
4-Methylcatechol	6.78, 6.66	-	-	-	↑	1.4	1.5
3,4-Dihydroxyphenylacetaldehyde (DOPAL)	6.74, 6.70,	↑	1.2	1.6	↑	1.3	1.7
Unknown	9.62	-	-	-	↑	1	2.1
2,2-Dimethylsucssinic acid (2,2-DMS)	2.66	↓	2.1	3.6	↓	1.3	4.5
Beta-alanine	2.54	↓	1.8	2.4	↓	1.3	3.5
Nicotinamide ribotide (NMN)	8.98	↑	1.13	1.5	-	-	-
Nicotinamide	8.90	↑	1.16	1.5	-	-	-
Nicotinamide adenine dinucleotide (NAD)	8.38	↑	1.5	1.5	-	-	-
Nicotinic acid	8.94	↑	1.1	1.6	-	-	-
Guanosine triphosphate (GTP)	6.74, 8.14	↑	1.2	1.5	-	-	-
Epi-coprostanol	0.5, 0.46, 0.22, 0.26,	↓	1.3	2.1	-	-	-
Pyridoxine	2.5	↓	1.7	2.3	-	-	-
Hippuric acid	7.82, 7.54, 3.94	↓	1.5	1.6	-	-	-
Anthranilic acid	7.78	↓	1.8	1.6	-	-	-
Unknown	9.94	↓	1.3	1.5	-	-	-

Notes: ↑ represents overrepresentation of the metabolite in the urine of LN patients; ↓ represents underrepresentation of the metabolite in the urine of LN patients; VIP: variable importance in the projection; LN: lupus nephritis; HC: healthy control

**Table 4:** Receiver operating characteristic (ROC) analysis of potential lupus nephritis (LN) biomarker candidates

**Biomarkers**	**AUC**	**Sensitivity (%)**	**Specificity (%)**
4-Methylcatechol	0.73	71%	82%
3,4-Dihydroxyphenylacetaldehyde (DOPAL) ^(c)^	0.72 (LN vs HC) 0.71 (LN vs SLE)	71% (LN vs HC) 71% (LN vs SLE)	82% (LN vs HC) 70% (LN vs SLE)
Unknown (9.62 ppm)	0.72	71%	64%
2,2-Dimethylsucssinic acid (2,2-DMS ) ^(b)^	0.87 (LN vs HC) 0.88 (LN vs SLE)	90% (LN vs HC) 80% (LN vs SLE)	79% (LN vs HC) 100% (LN vs SLE)
Beta-alanine ^(a)^	0.9 (LN vs HC) 0.85 (LN vs SLE)	90% (LN vs HC) 80% (LN vs SLE)	100% (LN vs HC) 86% (LN vs SLE)
Nicotinamide ribotide (NMN)	0.73	100%	60%
Nicotinamide	0.74	79%	60%
Nicotinamide adenine dinucleotide (NAD)	0.79	100%	60%
Nicotinic acid	0.73	100%	60%
Guanosine triphosphate (GTP)	0.74	79%	60%
Epi-coprostanol	0.68	70%	64%
Pyridoxine	0.80	80%	100%
Hippuric acid	0.76	70%	99%
Anthranilic acid	0.74	70%	86%
Unknown (9.94 ppm)	0.61	60%	65%
a + b	0.87	76%	100%
a + c	0.89	76%	100%
b + c	0.89	81%	100%
a + b + c	0.89	81%	100%

a, b, and c represent Beta-alanine, 2,2-dimethylsucssinic acid, and 3,4-Dihydroxyphenylacetaldehyde (DOPAL), respectively

**Table 5:** List of contributed pathways in the pathogenesis of lupus nephritis (LN). All encompassed overlapping metabolites in these pathways belonged to metabolites differentiated between LN and systemic lupus erythematosus (SLE) groups

**Pathway name**	**Pathway source**	**Number of overlapping metabolites**	**P- value**	**Corrected ** ***P*** **-value**
Nicotinate nicotinamide metabolism	INOH	4	1.18 × 10 ^-7^	0.000433
DNA damage recognition in GG-NER	Reactome	2	3.71 × 10 ^-6^	0.00156
Degradation of AXIN	Reactome	2	3.71 × 10 ^-6^	0.00156
Downregulation of SMAD2/3:SMAD4 transcriptional activity	Reactome	2	2.22 × 10 ^-5^	0.0072
SIRT1 negatively regulates rRNA expression	Wikipathways	2	2.22 × 10 ^-5^	0.0072
HDR through MMEJ (alt-NHEJ)	Reactome	2	3.70 × 10 ^-5^	0.0082
Regulation of PTEN stability and activity	Reactome	2	3.70 × 10 ^-5^	0.0082
DNA repair	Reactome	3	5.61 × 10 ^-5^	0.00876
Regulation of HSF1-mediated heat shock response	Reactome	2	5.54 × 10 ^-5^	0.00876
Transcriptional activity of SMAD2-SMAD3-SMAD4 heterotrimer	Wikipathways	2	5.54 × 10 ^-5^	0.00876
POLB-Dependent Long patch base excision repair	Reactome	2	5.54 × 10 ^-5^	0.00876
Resolution of AP sites via the multiple-nucleotide patch replacement pathway	Reactome	2	5.54 × 10 ^-5^	0.00876
Transcriptional activation of mitochondrial biogenesis	Reactome	2	5.54 × 10 ^-5^	0.00876
Signaling by TGF-beta Receptor Complex	Reactome	2	7.75 × 10 ^-5^	0.0105
Cellular response to heat stress	Reactome	2	0.000103	0.0112
Resolution of abasic sites (AP sites)	Wikipathways	2	0.000103	0.0112
Processing of DNA double-strand break ends	Reactome	2	0.000103	0.0112
tRNA splicing	HumanCyc	2	0.000133	0.0127
Negative epigenetic regulation of rRNA expression	Reactome	2	0.000166	0.0145
&beta;-alanine degradation	HumanCyc	2	0.000202	0.0167
GABA synthesis_ release_ reuptake and degradation	Wikipathways	2	0.000333	0.0255
dopamine degradation	HumanCyc	2	0.000497	0.0355
guanosine nucleotides de novo biosynthesis	HumanCyc	2	0.000497	0.0355
PIP3 activates AKT signaling	Reactome	2	0.000692	0.047

## Conclusions

The present study provided proof of concept that the ^1^H-NMR based urine metabolomics approach has high sensitivity and specificity to discriminate LN from SLE and HC cohorts.

The present study investigated the urine metabolic profile of LN patients using ^1^H-NMR based metabolomics approaches, and a panel of three candidate biomarkers was identified.

Different relevant pathways were identified based on differential metabolites that are involved in the pathogenesis of LN. These pathways and the genes related to differential metabolites are potential targets for future analyses to control the renal complications of SLE. 

Our findings revealed the efficiency of urine metabolomics in the identification of potential biomarkers for LN and exploration of its underlying mechanisms. Thus, validation of these findings may contribute to the use of metabolic profile analysis in further clinical benefit.
